# Effect of video on satisfaction and self-confidence in simulation training: a randomized clinical trial

**DOI:** 10.1590/0034-7167-2022-0366

**Published:** 2023-06-26

**Authors:** Lissette Lucrecia Monge Abarca, Alba Lúcia Bottura Leite de Barros, Rui Carlos Negrão Baptista, Ruth Ester Assayag Batista, Juliana de Lima Lopes

**Affiliations:** I Universidade Federal de São Paulo. São Paulo, São Paulo, Brazil; II Escola Superior de Enfermagem de Coimbra. Coimbra, Portugal

**Keywords:** Simulation Training, Baths, Personal Satisfaction, Trust, Education, Nursing, Entrenamiento Simulado, Baños, Satisfacción Personal, Confianza, Educación en Enfermería, Treinamento por Simulação, Banhos, Satisfação Pessoal, Confiança, Educação em Enfermagem

## Abstract

**Objectives:**

to identify the effect on satisfaction and self-confidence of undergraduate nursing students after using a validated bed bath video during the simulation.

**Methods:**

blinded parallel randomized clinical trial. Participants were allocated to the control group (simulation with tutor) or intervention (simulation with video). After the interventions, the Student Satisfaction and Self Confidence with Learning Scale was used to assess satisfaction and self-confidence. The study was approved by the Ethics Committee and Brazilian Registry of Clinical Trials. Mann Whitney, Fisher Exact and Student t statistical tests were used. A significance level of 5% was adopted**. Results:** fifty eight students (30, control; and 28, intervention) were evaluated. There was no significant difference between the groups regarding satisfaction (p=0.832) and self-confidence (p>0.999).

**Conclusions:**

satisfaction and self-confidence were similar between the groups, and the two strategies could be used in the simulated practice of bed bathing.

## RESUMO

**Objetivos:**

identificar o efeito na satisfação e autoconfiança de estudantes do curso de graduação em Enfermagem após uso de um vídeo validado sobre o banho no leito durante a simulação.

**Métodos:**

estudo clínico randomizado paralelo e cego. Os participantes foram alocados no grupo-controle (simulação com tutor) ou intervenção (simulação com vídeo). Após as intervenções, utilizou-se a Escala de Satisfação dos Estudantes e Autoconfiança com a Aprendizagem para avaliar a satisfação e autoconfiança. O estudo foi aprovado pelo Comitê de Ética e Registro Brasileiro de Ensaios Clínicos. Foram utilizados os testes estatísticos Mann Whitney, Exato de Fisher e *t Student*. Adotou-se nível de significância de 5%.

**Resultados:**

avaliaram-se 58 estudantes (30, controle; e 28, intervenção). Não houve diferença significante entre os grupos quanto à satisfação (p=0,832) e autoconfiança (p>0,999).

**Conclusões:**

satisfação e autoconfiança foram similares entre os grupos, e as duas estratégias poderão ser utilizadas na prática simulada do banho no leito.

## RESUMEN

**Objetivos:**

identificar efecto en la satisfacción y autoconfianza de estudiantes del curso de grado en Enfermería tras uso de video validado sobre baño en el lecho durante el simulado.

**Métodos:**

estudio clínico randomizado paralelo y ciego. Los participantes fueron ubicados en grupo control (simulado con tutor) o intervención (simulado con vídeo). Tras intervenciones, utilizado la Escala de Satisfacción de Estudiantes y Autoconfianza con el Aprendizaje para evaluar satisfacción y autoconfianza. El estudio aprobado por el Comité de Ética y Registro Brasileño de Ensayos Clínicos. Utilizadas pruebas estadísticas Mann Whitney, Exacta de Fisher y *t Student*. Adoptado nivel de significación de 5%.

**Resultados:**

evaluados 58 estudiantes (30, control; y 28, intervención). No hubo diferencia significante entre los grupos cuanto a la satisfacción (p=0,832) y autoconfianza (p>0,999).

**Conclusiones:**

satisfacción y autoconfianza fueron similares entre los grupos, y las dos estratégias podrán ser utilizadas en la práctica simulada del baño en el lecho.

## INTRODUCTION

The bed bath is a procedure performed by the nursing team and is considered essential for the recovery of patients. It is indicated for bedridden individuals in permanent conditions, such as paralysis; or temporary, such as some surgeries or illnesses^(1)^.

When not performed in a safe and adequate manner, the bed bath procedure puts the patient’s physical integrity at risk, and may cause: accidents due to falls, accidental removal of devices, infections^(2)^, among other problems. Not knowing the body mechanics involved in performing this procedure can cause musculoskeletal injuries and diseases to those who perform it^(2)^. Therefore, nurses must know how to perform the technique correctly^(3)^.

The knowledge of the technique and the necessary skills to perform nursing procedures are taught from the undergraduate course. For some years, universities have been implementing new active teaching strategies, such as clinical simulation, which help in the development of clinical reasoning and in the training of technical skills, which contributes both to the safety of patients and future professionals^(4)^.

The simulation allows the partial or total reproduction of a real clinical situation in a safe and controlled environment; its application is related, in general, to practical activities that involve decision-making or skills training in certain procedures^(5)^. A wide variety of teaching tools can be used in conjunction with the simulation, and the use of educational videos is one of them.

The use of video is effective in acquiring cognitive capacity, improving retention of information and skills and can add satisfaction and self-confidence in learning^(6-9)^. In the simulation, satisfaction refers to contentment with learning through scenarios and can be an important component for self-confidence^(10)^. Self-confidence is related to the student’s confidence in their judgment and performance and can influence the translation of knowledge^(10)^.

Satisfied and self-confident students become more motivated to learn and tend to better master the contents and clinical skills taught to them; consequently, they become more capable of executing what they have learned in clinical practice^(10)^. Also, the knowledge of satisfaction and self-confidence allows assessing the scope of the proposed objectives, the performance and commitment of the students, in addition to being able to verify the quality of the teaching method adopted^(11-12)^.

In this sense, it is essential to identify teaching tools that increase these perceptions, because, in the literature, there is still a lack of studies that compared satisfaction and self-confidence in learning between different teaching strategies^(13)^.

## OBJECTIVES

To identify the effect on satisfaction and self-confidence of undergraduate nursing students after using a validated bed bath video during the simulation.

## METHODS

### Ethical aspects

The study was approved by the Research Ethics Committee of the university and registered in the Brazilian Registry of Clinical Trials - ReBEC (RBR-2tyxttk). All participants signed the Free and Informed Consent Form (FICF).

### Design, period, and place of study

This is a blinded parallel randomized clinical study, in which the Consolidated Standards of Reporting Trials (CONSORT) references were adopted. Data were collected in August 2021 at the Center for Teaching Skills and Simulation of a public university in the city of São Paulo, state of São Paulo (SP), Brazil.

### Population, inclusion, and exclusion criteria

The population consisted of all students aged over 18 years old and regularly enrolled in the second year of the undergraduate nursing course in the academic year of 2021 and who attended the Fundamentals of Nursing Care discipline. Those who were on sick leave during the data collection period and those who did not attend the theoretical class on bed bath were excluded.

### Study protocol

In the present study, this data collection procedure was followed: first, the students attended a dialogued expository class on bed baths, structured based on the literature. The class lasted approximately one hour and was taught by a professor of Fundamentals of Nursing Care, who was the tutor of the practical simulations.

After the class, the study subjects were asked about their desire to participate and make the data collected available for research, and those who agreed signed the FICF. After three days of theoretical class, students were invited to attend the Center for Teaching Skills and Simulation and were randomly allocated into one of two groups (intervention or control). Block randomization was performed using the Random^®^ System, with a total of ten blocks of six participants. The allocation was carried out by a professor (Researcher 1) who did not participate in the simulations or in the evaluations made by the students, that is, there was a blinding of this phase.

The intervention group (Group 1) consisted of students who watched the video, prepared and validated previously^(7)^, while practicing this procedure under the supervision of a tutor. The control group (Group 2) consisted of students who participated in the bed bath simulation without using the video, and the simulation of the procedure was performed under the guidance of a tutor. The participants of both groups performed the simulation in pairs, in a low-fidelity simulator, and the tutor of the control group and the intervention group was a professor of the Fundamentals of Nursing Care discipline (Researcher 2), previously trained in simulation. The bed bath simulation by both groups lasted up to 20 minutes.

After the simulated practice, the students were invited to move to another room to fill out the data collection instrument, consisting of two parts: the first contained closed questions about personal data (gender - female and male, age in complete years and experience prior execution of bed bath in clinical practice or teaching - yes or no); and the second was the Student Satisfaction and Self-Confidence with Learning Scale (SCLS), which was the outcome of the study. This instrument was applied by a student of the postgraduate course in Nursing, master’s level (Researcher 3), who was not aware of which group the student belonged to (control or intervention), thus maintaining the blindness of this phase.

The Student Satisfaction and Self-Confidence with Learning Scale was created in 2003 by the National League of Nursing (NLN)^(14)^. It consists of 13 items and two dimensions: the first measures student satisfaction with the simulation activity using five items. In the second dimension, self-confidence in learning is assessed using eight items. Both dimensions use a five-point Likert scale^(14)^. The scale’s reliability was tested using Cronbach’s alpha, in which a value of 0.94 was obtained for the “Satisfaction” dimension and 0.87 for “Self-confidence”^(13)^. It was translated and validated into Brazilian Portuguese in 2015 by Almeida et al.; and the analysis of internal consistency showed Cronbach’s alpha values of 0.94 for the construct “Satisfaction”; and 0.87 for “Self-confidence with learning”^(15)^.

### Analysis of results and statistics

The collected data were entered into a Microsoft Excel^®^ spreadsheet. Data entry was performed by the graduate student of the master’s course and checked by another researcher. Data analysis was performed by a statistician who was not informed which group represented the control group and intervention group. Qualitative data were presented in absolute (n) and relative (%) frequencies; and quantitative, in mean and standard deviation or median and interquartile range. To verify the homogeneity of the groups, regarding gender, age, and previous experience in performing the procedure, the Mann-Whitney and Fisher’s Exact tests were applied. The Anderson-Darling test was used to verify the normality of data distribution regarding satisfaction and self-confidence. The statistical tests used to compare students’ satisfaction and self-confidence between the control and intervention groups were Student’s t for repeated samples, if the distribution of variables showed normal distribution; or the Mann-Whitney test, if the distribution was not normal. P values less than 0.05 were considered statistically significant. All analyzes were performed using the free software R.

## RESULTS

**Figure 1:**
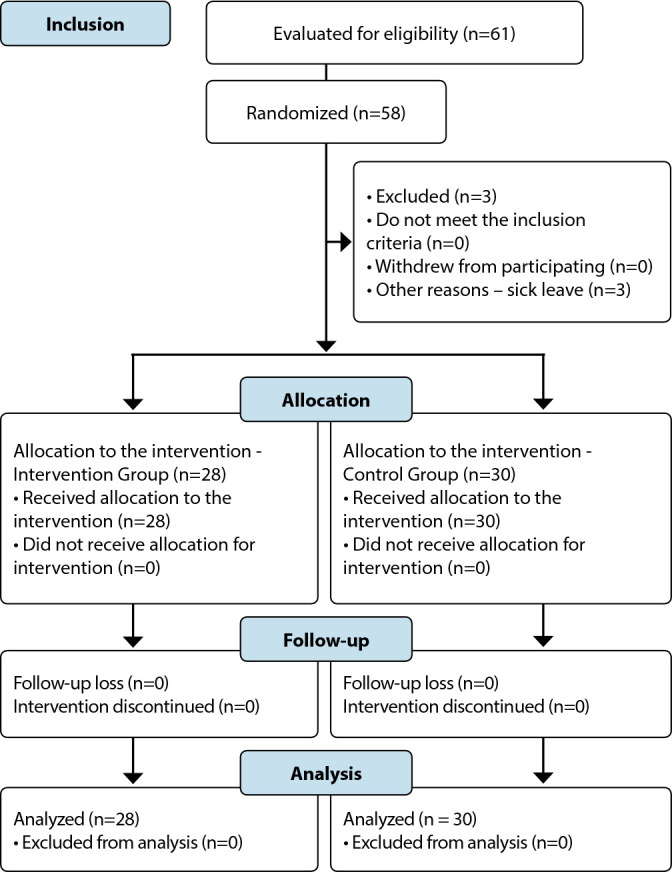
Clinical trial flowchart according to CONSORT 2010, São Paulo, São Paulo, Brazil, 2021

The sample consisted of 58 students, 28 from the intervention group and 30 from the control group (Figure 1).

In Table 1, it is observed that the groups were homogeneous in terms of age, gender, and previous experience of performing a bed bath.

**Table 1:** Age, gender, and previous experience of performing a bed bath for participants in the intervention and control groups, São Paulo, São Paulo, Brazil, 2021

Variables	Intervention group (n = 28) n (%)	Control group (n = 30) n (%)	*p* value
Sex			
Female	24 (85.71)	26 (86.67)	> 0.999^*^
Male	4 (14.29)	4 (13.33)	
Previous experience in performing a bed bath			
No	24 (85.71)	23 (76.67)	0.508^*^
Yes	4 (14.29)	7 (23.33)	
	**Mean** **(Standard** **deviation)**	**Mean** **(Standard** **deviation)**	
Age	21.43 (2.18)	21.8 (2.91)	0.85^†^

* *Fisher's Exact Test; †Mann-Whitney non-parametric test.*

In Table 2, it is seen that there was no statistical difference between the groups.

**Table 2:** Analysis of satisfaction and self-confidence between groups, São Paulo, São Paulo, Brazil, 2021

	General (n = 58) Mean (SD)	Intervention group (n = 28) Mean (SD)	Control group (n = 30) Mean (SD)	*p* value^*^
Satisfaction	23.41 (2.29)	23.43 (2.3)	23.4 (2.33)	0.832
Self confidence	35.59 (3.69)	35.64 (3.51)	35.53 (3.9)	> 0.999
General	59.0 (5.62)	59.07 (5.45)	58.93 (5.86)	0.95

* *Mann-Whitney non-parametric test; SD - Standard Deviation.*

## DISCUSSION

Teaching is constantly evolving. In this sense, adapting and updating teaching methods and tools are essential to ensure competent training of future professionals^(10)^. All teaching strategies have several characteristics that make them unique; and having clear the objective or expected results, as well as the target population, can guide the choice of relevant tools to be included in the simulation.

It is known that people have different abilities when it comes to learning. Some will find a particular teaching strategy to be a great way to learn; for others, it may be ineffective. Therefore, it is essential to investigate the different ways in which students learn, which methods and tools help in this process; and identify different learning patterns^(16)^.

There are still few studies that have analyzed the impact of using video associated with simulation. A Brazilian study analyzed: the effectiveness of using video associated with the simulation of port-a-cath catheter handling in anxiety; and the knowledge of 24 nursing students^(17)^. The students in this study watched the video three times before simulating the procedure on a mannequin, and the results showed that there was no difference in the level of anxiety before and after the intervention, but there was an increase in the number of correct answers after the video and execution of the simulated procedure (p = 0.000)^(17)^. The authors point out that this strategy is viable and useful to be implemented in the teaching of nursing students^(17)^.

A blinded, parallel randomized clinical trial showed that the use of video during the simulation increased the psychomotor skills of 56 second-year undergraduate Nursing students compared to the group that simulated the bed bath with guidance from a tutor (p. = 0.003)^(7)^.

Although the researchers point out that the use of video improves psychomotor skills, no research was identified that analyzed the effect of this tool on students’ satisfaction and self-confidence.

In the present study, it was observed that there was no difference between the simulation using the video and the one with a tutor in the level of satisfaction and self-confidence in learning. This result may be related to the fact that the simulated practices, regardless of the association of other tools, are carried out in a controlled environment, which can make the student more satisfied and self-confident - results, these, found by other researchers^(18-22)^.

A study carried out with 117 nursing students in Saudi Arabia showed that clinical simulation contributed to satisfaction (average of 3.76 to 4.0) and provoked a high level of self-confidence (average of 3.76 to 4.14) and motivation in the students^(18)^.

Another quasi-experimental study carried out in Brazil with 32 nursing undergraduate students, aimed at analyzing the effectiveness of simulation on self-confidence for out-of-hospital cardiopulmonary resuscitation, showed that this teaching strategy was effective in increasing nursing students’ self-confidence, to the execution of this emergency service. The results of this study revealed that there were statistically significant differences (p < 0.001) in the answers to all questions on the Self-Confidence Scale, when compared before and after the simulation^(19)^.

These same results were identified in a study carried out in the United States, with 61 students, in which simulation was used to teach community pediatrics for five weeks. The results showed that the average satisfaction and self-confidence of the students was 4.04+0.44. In the subscale “Satisfaction with learning”, the average score was 4.10+0.50, and the item that had the highest score was “The way the teacher taught was adequate for the way in which the student learns” (mean of 4.21+0.75). As for the subscale “Self-confidence in learning”, the results showed an average score of 4.00+0.46, and the item with the highest score was “It is my responsibility as a student to learn what I need to know through the simulation activity” (average of 4.26+0.72). The researchers of this study concluded that students were highly satisfied and self-confident with the teaching strategy and that simulation can connect theory with practice^(20)^.

Researchers from Spain analyzed the satisfaction of 91 nursing students in their second year of graduation regarding the simulated practice of initial patient assessment and monitoring of vital signs. In this study, the Simulated Clinical Experience Satisfaction Scale was used, consisting of 17 items and a ten-point Likert scale, where 1 indicates the lowest level of satisfaction: and 10, the greatest. The results indicated high levels of satisfaction when using this teaching strategy, with an overall score of 9.3^(21)^. In this same research, students described simulation as a playful learning method, allowing them to put their theoretical knowledge into practice and helping them to manage their fears before encountering real care environments^(21)^.

A Portuguese quasi-experimental study that analyzed the cognitive knowledge, satisfaction, and self-confidence of 94 Nursing students in the face of the simulation of a nursing consultation on vaccination showed that the use of the simulation not only improves knowledge compared to the traditional strategy (p < 0.000), but also promotes student satisfaction^(22)^.

Another quasi-experimental investigation, carried out in Turkey with 139 nursing students, whose objective was to compare the effect of different simulation modalities on levels of knowledge, skill, stress, satisfaction and self-confidence, showed high levels of satisfaction (mean of 4.01+ 0.85) and self-confidence (mean of 4.06+0.69) for those students who used a dummy in the simulation^(23)^ - scores similar to the present study.

In clinical nursing practice, satisfaction and self-confidence are essential factors because, in addition to helping students to complete their tasks accurately, they allow professionals to build a relationship of trust with their patients^(15,24)^. In this sense, the identification of teaching strategies through which students learn more, based on what motivates them and makes them confident, can help educational institutions in improving the training and preparation of future professional nurses^(10)^.

### Study limitations

As limitations of the study, the study was carried out in a single center and with a small sample. In this sense, the results found cannot be generalized to students with different characteristics from our population. Another limitation was the impossibility of analyzing the level of satisfaction and self-confidence before the interventions. However, it should be noted that the scale used is composed of questions that analyze these constructs after the simulation, not allowing their application before the interventions.

### Contributions to the field

The use of video can be an attractive and innovative tool to be used during the simulation. The method adopted in the present study can be replicated for other procedures, contributing to the advancement of teaching, and learning in nursing.

## CONCLUSIONS

There was no difference in the level of satisfaction and self-confidence in learning among undergraduate Nursing students when they simulated the bed bath with a tutor or with the video. Both strategies can be used to ensure satisfaction and self-confidence in the simulated bed bath practice.
